# Effectiveness and cost effectiveness of digital hearing aids in patients with tinnitus and hearing loss: a randomised feasibility trial (THE HUSH Trial)

**DOI:** 10.1186/s40814-022-01188-9

**Published:** 2022-11-03

**Authors:** Rachel H. Haines, Trish Hepburn, Wei Tan, Clare Jackson, James Lathe, Jennifer White, Christine Almey, Richard Nicholson, David Stockdale, Paul Leighton, Marilyn James, Magdalena Sereda

**Affiliations:** 1 Nottingham Clinical Trials Unit, School of Medicine, University of Nottingham, Nottingham, UK; 2 School of Medicine, University of Nottingham, Nottingham, UK; 3 PPI Representative, National Institute for Health and Care Research Nottingham Biomedical Research Centre, Nottingham, UK; 4 Nottingham University Hospitals NHS Trust, Nottingham, UK; 5 British Tinnitus Association, Sheffield, UK; 6 Centre of Evidence Based Dermatology, University of Nottingham, Nottingham, UK; 7 National Institute for Health and Care Research Nottingham Biomedical Research Centre, Nottingham, UK; 8 Hearing Sciences, Mental Health and Clinical Neurosciences, School of Medicine, University of Nottingham, Nottingham, UK

**Keywords:** Randomised controlled trial, Feasibility, Tinnitus, Hearing loss, Hearing aids, Outcomes

## Abstract

**Background:**

Education and advice is provided for tinnitus management in all UK audiology clinics. Sound therapy, including provision of hearing aids may be offered, but this is often dependent on a clinician’s decision rather than UK policy. This inconsistent management reflects a lack of evidence around the effectiveness of hearing aids for tinnitus. This open-label, two-arm multicentre randomised controlled feasibility trial gathered data around recruitment, acceptability and outcome assessments to determine the feasibility of conducting a large randomised controlled trial investigating the effectiveness of hearing aids for tinnitus management.

**Methods:**

Adults referred to audiology for tinnitus, with an aidable hearing loss were recruited at five UK audiology clinics. They were randomised 1:1 to either education and advice (treatment as usual (TAU), *n* = 41) or TAU plus hearing aids (*n* = 42). Outcomes were collected by questionnaires 12 weeks after randomisation. After participation, interviews were conducted with a subset of both participants and clinicians from each trial centre.

**Results:**

Eighty three participants from five sites were randomised. Non-aidable hearing loss was the main reason for ineligibility to participate in the trial reported by the sites. Seventy three percent of participants returned the 12-week questionnaires, with return rates by site ranging from 61 to 100%. Fifteen out of 33 participants (45%) reported using hearing aids for the clinician-recommended time, or longer, during the day. The Tinnitus Functional Index (TFI) was the outcome measure most responsive to change. The majority of participants also agreed it was relevant to their tinnitus and hearing loss. Qualitative data demonstrated that the trial was acceptable to participants. Feedback from clinicians revealed a potential lack of equipoise. It also highlighted the differences in referral and treatment pathways between departments and differences in audiometric criteria for fitting hearing aids. Health economic measures were well completed for those returned. No change in health-related quality of life was observed. Costs were higher in the intervention arm, but self-reports of healthcare service use indicated participant confusion in treatment pathways.

**Conclusions:**

This feasibility trial is the first step towards obtaining high quality evidence to determine potential clinical effectiveness and cost effectiveness of hearing aids for tinnitus versus usual care. A definitive trial was deemed to be feasible, with some modifications based on feasibility findings and using the TFI as the primary outcome.

This trial was funded by the National Institute for Health Research, Research for Patient Benefit Programme (PB-PG-0816–20,014) and registered with ISRCTN (ISRCTN14218416).

**Supplementary Information:**

The online version contains supplementary material available at 10.1186/s40814-022-01188-9.

## Key messages regarding feasibility

- This trial gathered data around recruitment, acceptability and outcome assessments to determine the feasibility of conducting a large randomised controlled trial to determine the effectiveness of hearing aids for tinnitus management.
- A definitive RCT is feasible, providing design optimisation based on the feasibility findings are introduced into the design of future trials and using the Tinnitus Functional Index as the primary outcome.
- The design of the definitive trial will need to take into consideration current differences between audiology departments in terms of referral and treatment pathways and hearing aid fitting criteria, and make improvements on the quality of information given to participants at time of recruitment.

## Introduction

Tinnitus, the awareness of a sound in the absence of an external source, is a major problem affecting 10–30% of the adult population [1]. It is thought to result from abnormal activity in the auditory pathway. About 20% of people with tinnitus experience symptoms that negatively impact on their quality of life (sleep disturbances, hearing difficulties, difficulties with concentration, social isolation and emotional difficulties including anxiety, depression, irritation or stress), requiring clinical intervention [2]. It is also estimated that tinnitus prevalence in people with hearing loss is as high as 70–85%, with the incidence of clinically bothersome tinnitus increasing with age [3].

Tinnitus is associated with pathological neural activity in the central auditory system, possibly resulting from a maladaptive response to a peripheral lesion (hearing loss); several structures in the central auditory system, such as the dorsal cochlear nucleus, inferior colliculus, and auditory cortex have been implicated as possible sites for tinnitus generation [4]. Animal studies have observed physiological changes in these structures following noise-induced trauma, including hyperactivity, increased synchronous activity, and functional reorganisation (change in the response properties of neurons to external sounds) [5–7]. It has also been postulated that spontaneous hyperactivity could lead to increased central ‘gain’ in the auditory cortex in response to reduced auditory input. Whilst increased gain would lead to stabilising mean firing and neural coding efficiency, it would also amplify ‘neural noise’, resulting in tinnitus generation [8–10]. Moreover, both animal and human studies point to the involvement of non-auditory brain regions, associated with cognition and emotional processing, in the pathophysiology of tinnitus [11, 12].

Both the prevalence and impacts on quality of life, tinnitus represents a significant burden on healthcare services. An estimated 0.75 million primary care consultations where tinnitus is the primary complaint take place each year in the England alone [13]. The annual NHS tinnitus expenditure and the annual societal costs were estimated to be GB£750 M and £2.7 billion respectively [14]. Approximately 90% of those presenting with tinnitus also have hearing loss and are thus potentially eligible for fitting of hearing aids [2, 15, 16]. The majority have mild hearing loss, representing the population where clinical practice is the most variable. And though the National Institute for Health and Care Excellence tinnitus guideline recommends provision of hearing aids for people with perceived hearing difficulties, it lacks specific recommendations on the provision of hearing aids for tinnitus [17].

In the UK, the most common management strategy for patients presenting with bothersome tinnitus is education and advice. This typically includes explanations of the aetiology of tinnitus and its association with hearing loss, education about lifestyle factors that may have positive or negative effects on the perception of tinnitus and a description of available management strategies [18]. Some audiologists may also combine this advice with sound therapy, such as hearing aids or sound generators [19]. However, there is no standard care pathway in the UK, and variability in provision of such devices is dependent on clinical decisions which are themselves influenced by the experiences or personal opinions of the treating audiologist or local guidelines [20].

The primary function of a digital hearing aid is to amplify and modulate sound to make it more accessible and to aid communication. Using hearing aids in tinnitus management has been proposed since the 1940s [21]. However, the benefit reportedly varies and there is no clear consensus on when a person would or would not benefit from amplification for their tinnitus [19, 22]. Several benefits of hearing aids for tinnitus have been proposed: amplifying environmental sounds and therefore masking or providing distraction from tinnitus, refocusing attention on alternative auditory stimuli that are incompatible and unrelated to the tinnitus sound, or reducing listening effort and improving communication which can in turn reduce the stress and anxiety commonly associated with tinnitus. Other possible mechanisms include physiological effects on tinnitus-related brain activity by recalibrating central gain or preventing maladaptive plastic changes in the brain related to hearing loss [23].

Despite these proposed benefits, there is no robust evidence or consensus on whether a person with tinnitus would or would not benefit from amplification in addition to the education and advice provided as standard care. A Cochrane review of sound therapy for the management of tinnitus cited a lack of high quality evidence leading to variability in clinical practice in the UK [23] and across Europe [24] and concluded that there is a need for high-quality evidence generated by rigorous methodology [23]. A survey of UK audiology departments has shown that half of the clinicians have different candidacy criteria for fitting hearing aids for patients with and without tinnitus [19, 20]. Patients with mild hearing loss and tinnitus are less likely to receive hearing aids than patients with more severe hearing losses, as are those who do not report hearing difficulties, despite the fact that tinnitus annoyance can be as debilitating in people who do not report hearing difficulties as in those who do [25, 26]. Evaluating the effectiveness of digital hearing aids for tinnitus was also identified as a priority research question for clinicians and patients in a James Lind Alliance Tinnitus Priority Setting Partnership [27]. As tinnitus can be debilitating for people regardless of severity of hearing loss, it is important to explore hearing aids in tinnitus management for anyone with an aidable loss.

A definitive trial assessing digital hearing aids for tinnitus is needed. However, there are few examples of multi-centre randomised controlled trials (RCT) in UK audiology clinics on which to inform the design and conduct of such a trial. As such, it was essential to first perform a feasibility trial. The HUSH trial (Hearing Aids for Tinnitus with Hearing Loss) was the first step towards a definitive trial to provide high quality evidence for the clinical effectiveness and cost effectiveness of hearing aid provision for tinnitus It investigated the feasibility and acceptability of a RCT comparing (i) education and advice (treatment as usual, (TAU)) and (ii) TAU plus digital hearing aids for the management of people with tinnitus and hearing loss.

Objectives around recruitment, outcome assessment, and acceptability were defined to (i) determine feasibility of a multicentre RCT comparing TAU and TAU plus hearing aids, (ii) optimise the design of a definitive RCT and (iii) determine which outcome measures are most relevant for patients to guide primary outcome selection for a definitive trial.

## Methods

The trial was registered prior to the start of recruitment (ISRCTN14218416, 8 August 2018). The previously published trial protocol provides full details of the study design and methods [28], and as such, these are only briefly described below. The reporting of this trial follows the Consolidated Standards of Reporting Trials (CONSORT) statement extension to randomised pilot and feasibility trials recommendations (Additional file 1) [29].

### Design

This was an open-label, two-arm, multicentre randomised (1:1) controlled feasibility trial comparing the effectiveness and cost effectiveness of ‘treatment as usual’ (TAU) to TAU plus hearing aids for the management of tinnitus in patients with hearing loss. The trial had a nested interview study to look at feedback from participants and clinicians regarding the trial and its intervention and also an economic evaluation to determine feasibility and appropriateness of tools for collecting resource use and health-related quality of life.

### Setting and participants

Adults (18 years or over) presenting to clinic with a primary complaint of tinnitus, as confirmed by the assessing audiologist, were recruited from five UK audiology departments. All participants provided written informed consent. Potential participants were excluded if their tinnitus was of a medically treatable origin, if they had used a hearing aid in the last 12 months or if they currently used any combination device or behind the ear sound generator, as these were considered to be concomitant treatments. Additional exclusions were the inability to communicate in written English, which would have prevented them from completing all outcome measures, and having started or stopped medication for anxiety or depression within the last 3 months, which could influence the efficacy of the interventions or the interpretation of changes in patient reported outcome measures (PROM).

### Participant recruitment and randomisation

From October 2018 to September 2019, potential participants were screened from tinnitus clinic lists and sent a participant information sheet in the post providing information about the trial. During their routine care appointment, the patient was approached to take part in the study by the audiologist if they were deemed eligible. After written informed consent was given, audiometry and the Tinnitus Functional Index were first undertaken to provide the necessary minimisation variables (trial site, tinnitus severity and degree of hearing loss) for randomisation (1:1). The local site randomised the participant using a concealed, secure web-based system developed by the NCTU and held on a secure UoN server.

### Trial intervention

After randomisation, both groups received education and support (TAU) according to local practice. TAU could not include any ear-level device such as an at-ear sound generator. The intervention group also received hearing aids which were fitted according to standard procedures. This had to be fitted either on the same day as randomisation or within a 4-week window to allow for trial sites where the patient pathway did not allow for hearing aid fitting on the same day as the assessment appointment. At the time of fitting the audiologist gave daily usage recommendations. Adherence was checked at the end of the 12-week treatment phase, comparing participant reported usage in the questionnaire to this clinician-recommended usage.

### Blinding

As hearing aids were offered only to the intervention group, blinding of treatment allocation was not possible for the clinician or the participant. Members of the trial management team were also unblinded for purposes of questionnaire administration. The trial statistician, health economist and chief investigator remained blinded to all treatment allocations until after database lock.

### Outcome measures

Outcomes to determine the feasibility and acceptability of a large definitive trial were collected. A range of PROM was also gathered to inform the choice of primary outcome for a definitive trial.

#### Feasibility and acceptability

1. *Recruitment*: number and proportion of eligible and randomised patients; patient characteristics; adherence; retention; data completeness; components of TAU; hearing aid fitting parameters; re-referral rates; feasibility of collecting healthcare resource use and safety data. 2. *Acceptability*: hearing aid adherence; feedback from participants and clinicians regarding trial conduct and design as well as the intervention. 3. *Outcomes*: participant opinion of PROM; distribution of PROM; estimation of clinically important differences of PROM.

#### PROMs

The following PROMs were collected at baseline and 12 weeks: tinnitus symptom severity (Tinnitus Functional Index (TFI) [30]), hearing impairment effects on emotional and social adjustment (Hearing Handicap Inventory for the Elderly (HHIE) [31]), health status and quality of life (Health Utilities Index Mark 3 (HUI3) [32]), Depression and Anxiety (Hospital Anxiety and Depression Scale (HADS) [33]), measurement of tinnitus symptoms most important to the patient (Measure Yourself Medical Outcome Profile (MYMOP2) [34]), quality of life (EuroQoL-5D five level version (EQ-5D-5L) [35]), and Global Rating of Change (Clinical Global Impression of perceived change in tinnitus/hearing), as determined by asking two questions: all things considered, how is your overall [(1) tinnitus (2) hearing] now, compared to 3 months ago? This was rated on a 7-point Likert scale: ‘much improved’, ‘moderately improved’, ‘slightly improved’, ‘no change’, ‘slightly worse’, ‘moderately worse’ and ‘much worse’. Participant opinion on the relevance of each measure was also collected at 12 weeks when participants were asked to rate how relevant each questionnaire was with respect to their tinnitus and hearing loss on a 5-point scale, ranging from strongly agree to strongly disagree.

#### Outcome collection

PROMs were collected at baseline appointments and in 12 week-follow-up questionnaires between November 2018 and December 2019, using paper questionnaires either given or sent directly to the participants. Data was entered into the eCRF upon return of the questionnaires to the trial coordinating centre. Non-return of the questionnaires was followed up using a combination of post, email, text and telephone reminders. Recruitment outcomes were taken from data collected and entered by clinicians during the running of the trial at site, and qualitative data was collected from interviews with participants and clinicians after the end of their individual involvement in the trial.

#### Sample size

Since this was a feasibility trial, a formal calculation was not appropriate. The trial aimed to randomise 100 participants over 12 months from five recruiting centres to explore feasibility parameters such as methods of recruitment and recruitment and retention rates in different types of centres. This sample size enabled estimation of a retention rate of greater than 80% to within 8% points of the true value with 95% confidence. Together with information from qualitative interviews and the information collected about PROMs which will inform the primary outcome and sample size for a future trial, this provided sufficient to determine feasibility for a future definitive trial. The five sites were each given a target of randomising 20 participants.

#### Statistical analyses

The trial was analysed in accordance with a full statistical analysis plan, finalised prior to database lock and release of treatment allocations. Formal statistical testing and interim analyses were not undertaken as the aims of this trial were to assess feasibility. Descriptive summaries were produced for total and site recruitment rates, the number and proportion of participants who underwent each allocated procedure, completeness of data collection at baseline and 12 weeks by trial arms and outcome data at baseline and 12 weeks.

#### Analyses for selection of potential primary outcome

To guide the choice of primary outcome for a definitive trial, analyses of PROMs, independent of treatment group, were performed and taken into consideration alongside patient views captured in the nested interview study (described below).

For each of the five PROMs, total scores at baseline and 12 weeks, and change from baseline to 12 weeks were summarised. Scores for participants who answered ‘slightly improved’ or ‘moderately improved’ on the global improvement questions were used as an anchor to investigate the smallest difference that participants perceived to be beneficial. Minimum clinically important effects for each PROM was estimated using three standard anchor-based responsiveness statistics [36]: (i) standardised response mean (SRM), (ii) effect size (ES) and (iii) Guyatt’s Responsiveness Index (GRI).

#### Nested interview study

A nested interview study took place alongside the trial to assess the feasibility and acceptability of trial processes and the intervention and to further consider the patient perspective in regard to PROMs. Upon completion of individual participation in the trial, semi-structured telephone interviews were conducted with both study participants and healthcare professionals representing all study centres. Participants were be selected purposively to capture a range of different experiences and treatment outcomes. After recruitment activities have ceased, clinical staff at each study site was interviewed to review their experience of the trial in terms of participant recruitment and integrating the research within clinical care. Interviews were recorded, transcribed verbatim in accordance with the study protocol and analysed using a framework approach [37, 38] to support recommendations for the design of the definitive trial.

#### Health economics

A trial-specific proforma was developed to assess the feasibility of capturing healthcare resource use, and the appropriateness of the health-related quality of life (HRQoL) instruments, for remote data collection in this population. Measures were taken at baseline (HRQoL) and at 12 weeks (HRQoL + resource use). The purpose-built questionnaire, which collected data on healthcare resource use, took an NHS and societal perspective and included variables for trial attributable service and device use, such as audiologist/hearing therapist contacts, alongside other external pathways such as primary and community services. Applicable unit costings were derived from the National Cost Collection [39] and the Unit Costs of Health and Social Care [40], shown in Table A3. Private services and devices used participant self-reports where available. Health state utilities were derived from the EQ-5D-5L using a 5L- > 3L mapping tool [41] and UK valuation set [42], the HUI3 is a North American measure and as such applied a Canadian tariff [43]. Quality adjusted life-years (QALYs) were constructed using area under the curve (AUC), adjusted through baseline regression [44] and point estimates reported alongside 95% confidence intervals (bootstrapped percentile) [45]. Analyses included data completeness, descriptive statistics and mean differences of QALYs and costs between our arms.

#### Trial oversight and assessment of feasibility

The trial management group (TMG) met on a monthly basis over the course of the trial, and an independent trial steering committee (TSC) maintained regular overall oversight for the conduct of the trial. The TMG and the TSC took a joint decision on final assessment of feasibility using both the quantitative and qualitative data gathered over the course of the trial.

The feasibility assessment was adapted from that proposed by Thyer et al. [46]. The trial would be deemed successful if (1) 80% of recruitment target achieved; (2) number of sites retained, number of site achieving recruitment target, study consent and retention rates and proposed sample sizes, indicate delivery of the full RCT is plausible within a 5-year study period and (3) participants and clinicians report acceptability of the trial.

## Results

### Recruitment

**Fig. 1:**
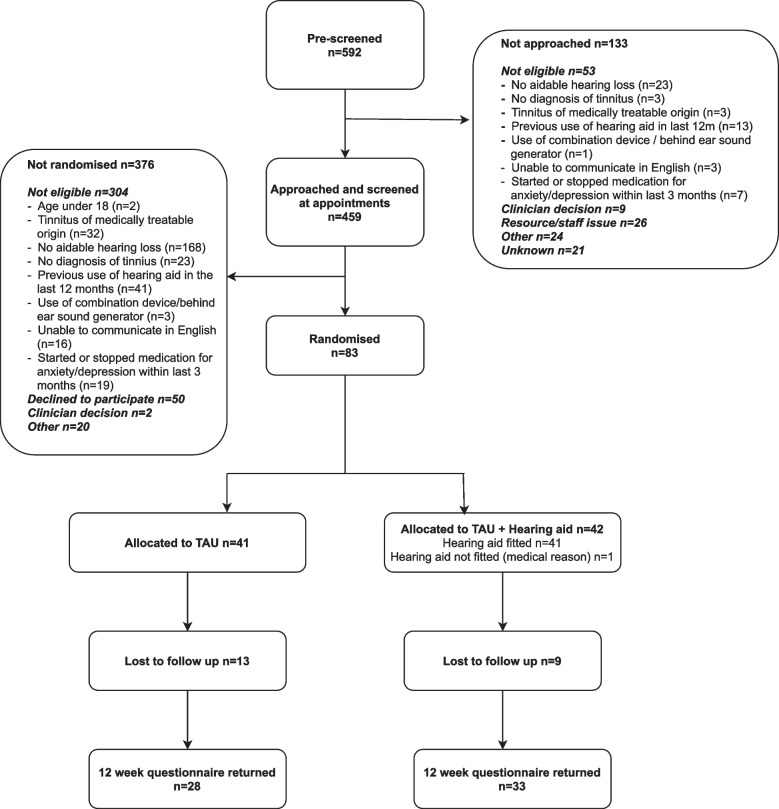
Participant flow diagram

Five sites were open for recruitment between 15 October and 3 December 2018. All sites closed to recruitment on 30 September 2019. During the recruitment period, 1737 patients were referred to these centres for tinnitus. Of those, 592 (34%) were pre-screened for trial eligibility, either prior to appointment based on existing medical records or at the appointment itself: 155 (26%) were eligible to participate in the trial (Fig. 1). The most common reasons for ineligibility were non-aidable hearing loss (i.e. hearing loss not meeting criteria for fitting hearing aids at study site; *n* = 191, 32%), previous use of hearing aids in the last 12 months (*n* = 54, 9%), no diagnosis of tinnitus (*n* = 26, 4%) and starting or stopping medication for anxiety/depression in the last 3 months (*n* = 26, 4%).

Eighty-three of the 155 (54%) eligible patients were randomised (41 to TAU and 42 to treatment as usual with digital hearing aids). Three out of five sites met or exceeded their recruitment target of 20 participants (Table 2).

### Baseline characteristics

Baseline demographics, clinical characteristics (Table 1) and PROMs (Table A1) were balanced between the two treatment arms. Participants reported a range of tinnitus sounds, with the most common sounds reported being buzzing, ringing, hissing, whooshing, tonal and whistling (all > 15% of participants, not mutually exclusive). Seventy-two (87%) reported their tinnitus as being non-pulsatile.

**Table 1:** Demographic and clinical characteristics

	**TAU (*n* = 41)**	**TAU + Hearing aid (*n* = 42)**	**Total (*n* = 83)**
**Age at randomisation (years)**			
Mean [SD]	61.1 [10.8]	55.4 [15.6]	58.2 [13.6]
**Duration of tinnitus (years)**			
Mean [SD]	7.0 [8.3]	8.7 [10.4]	7.8 [9.4]
**Gender**			
Male	24 (59%)	27 (64%)	51 (61%)
Female	17 (41%)	15 (36%)	32 (39%)
**Ethnicity**			
White	40 (98%)	39 (93%)	79 (95%)
Black Caribbean	0	3 (7%)	3 (4%)
Sikh	1 (2%)	0	1 (1%)
**Degree of hearing loss**^a^			
Mean [SD]	41.2 [17.3]	39.2 [13.4]	40.2 [15.4]
**Severity of tinnitus (Tinnitus Functional Index score)**^b^			
Mean [SD]	57.1 [21.9]	57 [22]	57.1 [21.8]
**Initial onset of tinnitus related to**^c^			
Change in hearing	3 (7%)	0	3 (4%)
Whiplash	0	1 (2%)	1 (1%)
Stress	2 (5%)	2 (5%)	4 (5%)
Head trauma	0	1 (2%)	1 (1%)
Loud sound	0	2 (5%)	2 (2%)
Other cause	13 (32%)	7 (17%)	20 (24%)
Unknown	23 (56%)	29 (69%)	52 (63%)
**Location of tinnitus**			
Bilateral	25 (61%)	28 (67%)	53 (64%)
Unilateral	9 (22%)	11 (26%)	20 (24%)
Inside the head	7 (17%)	3 (7%)	10 (12%)
**Tinnitus sound over time**			
Intermittent	9 (22%)	7 (17%)	16 (19%)
Constant	32 (78%)	35 (83%)	67 (81%)
**Sounds of tinnitus**^c^			
Hissing	6 (15%)	13 (31%)	19 (23%)
Ringing	10 (24%)	9 (21%)	19 (23%)
Pulsing	1 (2%)	2 (5%)	3 (4%)
Buzzing	11 (27%)	11 (26%)	22 (27%)
Clicking	2 (5%)	1 (2%)	3 (4%)
Cracking	0	2 (5%)	2 (2%)
Tonal	9 (22%)	5 (12%)	14 (17%)
Humming	5 (12%)	5 (12%)	10 (12%)
Popping	1 (2%)	1 (2%)	2 (2%)
Roaring	0	3 (7%)	3 (4%)
Rushing	2 (5%)	1 (2%)	3 (4%)
Whistling	5 (12%)	8 (19%)	13 (16%)
Whooshing	6 (15%)	9 (21%)	15 (18%)
Other	7 (17%)	11 (26%)	18 (22%)
**Tinnitus seem to pulsate**			
Pulsatile	4 (9%)	5 (12%)	9 (11%)
Non-pulsatile	37 (90%)	35 (83%)	72 (87%)
Do not know	0	2 (5%)	2 (2%)

All data are N (%)’s unless specified

^a^Based on the average values of 6 pure-tone hearing threshold levels at 500, 1000, 2000, 4000, 6000 and 8000 Hz. This was different from guidelines of British Society of Audiology and was chosen to include participants with hearing loss at higher frequencies.

^b^Derived from baseline TFI score: 0–28 = mild, 29–65 = moderate, >  = 65 = severe [47]

^c^Not mutually exclusive

Fifty-one (61%) men and 32 (39%) women were randomised with a mean age of 58 (SD = 13.6) years. Seventy-nine (95%) participants were white. Median duration of tinnitus was 3.8 years (range 0.4–40 years).

### Compliance with allocated treatment

Eighty-two participants received their allocated treatment. One participant randomised to receive hearing aids was not fitted due to medical reasons identified after randomisation. Twenty-four participants received bilateral (59%) and 17 (41%) unilateral hearing aids.

Fifteen out of the 33 participants who returned their end of trial questionnaires (45%) reported using hearing aids for the time recommended by the clinician or longer during the day (during weeks 11 and 12). Thirteen participants (39%) reported using hearing aids for less time during the day than recommended by clinicians. Five participants (15%) reported that they were not wearing their hearing aids at all during weeks 11 and 12. Reported reasons for non-use were as follows: (i) pain when using, (ii) made no difference to tinnitus, (iii) did not get used to the HAs, (iv) ear dryness needing treatment and (v) HAs made them more aware of tinnitus.

### Retention and follow-up

Sixty-one participants (73%) returned the 12-week questionnaire packs [28/41 (68%) in the TAU group and 33/42 (79%) in the TAU plus hearing aids group. Return rates ranged from 61 to 100% between different trial sites (Table 2).

**Table 2:** Recruitment and retention by trial site

Site	Number participants randomised	Number of 12 week questionnaire returned
1	20	15 (75%)
2	2	2 (100%)
3	31	19 (61%)
4	10	9 (90%)
5	20	16 (80%)
**All site**	**83**	**61 (73%)**

### Components of TAU

TAU was similar between two treatment arms (Table 3). The fundamental components of TAU were also similar across all sites: information and education about tinnitus and informal counselling was provided to 82 (99%) of participants. In general, the majority of participants also received advice on mobile applications, stress, sleep management and relaxation techniques.

**Table 3:** Summary for treatment as usual by arm (all randomised participants)

Treatment option	Control (*n* = 41)	Intervention (*n* = 42)
Information and education about tinnitus	41 (100%)	42 (100%)
Informal counselling	41 (100%)	41 (99%)
Formal counselling	1 (2%)	3 (7%)
External sound generators	22 (54%)	22 (52%)
Mobile phone/device software or applications	36 (88%)	38 (91%)
Advice on stress	37 (90%)	36 (86%)
Advice on sleep management	36 (88%)	34 (81%)
Advice on relaxation techniques	39 (95%)	35 (83%)
Other	2 (5%)	1 (2%)

Some between site differences were noted: in particular, one centre offered external sound generators to the majority of participants (94 and 100%) whereas others offered them less frequently (45, 15, and 10%).

Sites were also questioned about re-referrals, to explore the mechanisms for capturing and using these data in a definitive trial. However, at the time of asking, trial participants had not yet been or had only been recently discharged from clinics, so there were no re-referral data.

### Outcome completion

Data collected in the clinic on the components of TAU and about hearing aid fitting were 100% complete. At baseline 100% of TFI, HHIE, and HADS questionnaires, 99% of HUI3, 95% of MYMOP2, and 99% of EQ-5D-5L questionnaires were fully completed. Of the 61 questionnaires returned at 12 weeks, data completeness for PROMs was between 92 and 100%. Data completeness were similar between treatment groups.

### PROM assessment

Eighty-four percent of participants (75% in the TAU and 91% in the TAU + hearing aid arm) either strongly agreed or agreed at the end of the trial that the TFI was relevant to their tinnitus and hearing loss. Overall percentages of ‘strongly agree’ or ‘agree’ on relevance were lower for all other PROMs. There was no evidence of floor or ceiling effects for all PROM scores at baseline and 12 weeks.

TFI and MYMOP were more responsive to change than HHIE and HADS scores with Guyatt’s responsiveness indices of − 0.90, − 0.67, − 0.54 and 0.34, respectively. Within participant MCIDs based on the data for the trial participants were − 16 for TFI, − 8.3 for HHIE, 1.9 for the HADS overall score and − 0.7 for MYMOP2.

### Treatment outcomes

There appeared to be a greater change from baseline in the TAU + hearing aid group in all PRO scores at 12 weeks (Table A1). The largest relative differences between groups were shown for the TFI overall score and the HHIE.

More participants reported that their tinnitus and hearing had either ‘much’ or ‘moderately’ improved at 12 weeks in the TAU + HA arm than the TAU arm (11/33 (33%) vs 4/28 (14%)).

### Safety

A small proportion of participants (10/61 (16%)) had at least one of worsening of tinnitus, worsening of hearing, development of anxiety or development of depression at 12 weeks. This was similar between treatment arms ((5/28 (18%) in TAU arm and 5/33 (15%) in TAU + HA)). One participant was admitted to hospital during the 12-week treatment period, which was unrelated to trial interventions.

### Nested interview study

Twenty-three trial participants and 10 healthcare professionals were interviewed; each study centre was represented by at least one trial participant and one healthcare professional. Data presented here reports both participants’ and healthcare professionals’ experience of the research processes, and their assessment of the potential for further tinnitus research.

Study participants were positive about the potential for further research in this topic and were largely uncritical about the form and requirements of this and any further study. Some were unclear about the purpose of this study and unsure about their duration of involvement—but this was not a source of anxiety or concern for them (such was their positivity about the value of tinnitus research).

Participant 6: If you are not fully aware of what and why a project has been introduced, then there is no point in trying to guess or get involved in that aspect, people far cleverer than I have decided to do this HUSH project and I’m just one of the many, I would think, who are here to help.

Some participants found the length of follow-up instruments off-putting, and some were unclear about the purpose of these instruments—no longer completing them when they had ceased to use their hearing aid. With this exception, participants were generally positive about what they were required to do in the study.

Participant 17: The fact that I didn’t wear the [hearing aid put me off filling it out] … I looked through the questionnaire and there was nothing to say ‘did you actually wear the hearing aids when you are supposed to?’, so I was like that’s a bit silly because I actually didn’t wear the hearing aids so I didn’t want to fill it out to look like I’d got no benefit but that would have been because I didn’t wear the hearing aids for 12 weeks […] so I didn’t want to give false results.

Participant 16: Some of the questions were a little bit repetitive but written in different ways. I can’t actually give you an example at this moment in time but I did find that I seemed to be answering the same thing again.[…] it might have been there to see if well maybe like a bit of a trick question, maybe if you answered one one way and one another way then like are you really at the right answer.

The potential value of a hearing aid for tinnitus was not universally recognised by all participants, with some assuming that the provision of a hearing aid was primarily for their hearing loss rather than for their tinnitus. Some participants experienced technical issues with their hearing aids, and some struggled to acclimatise to them.

Participant 6: When she said I’m going to give you a hearing aid I thought that would improve my hearing but it hasn’t and in actual fact I don’t know if I should say this or not, I don’t think it’s working. [A friend] said, she asked me ‘I see you’ve got a hearing aid’, I said, ‘yes but I don’t think it’s working’ and I told her all about what had happened and I said, ‘but I’m going to [City] on Monday to see my daughter I was thinking of calling in the [Hospital]’ and she said, ‘well I think you have got to accept that they may have given you a placebo’.

Healthcare professionals highlighted different local referral and treatment pathways and indicated that incorporating the research processes into routine clinical practice was difficult and a possible barrier to effective recruitment.

Professional 8: We used our direct referral clinics as the targeted patients for the study and I think that’s why our recruitment numbers were low because essentially a lot of those patients needed medical opinion or needed some intervention before we could go on to do the standard care or the hearing aid and some of them would have normal hearing because they had just been referred in for tinnitus so actually we found it quite difficult to recruit into the study because they were coming in blind we had no idea of whether they were going to have hearing loss or whether they were going to be suitable to be recruited into the study.

It was also clear that differences in local/professional practice influenced how professionals engaged with the recruitment process—where they already routinely recommended hearing aids there was some anxiety about withholding treatment.

Professional 4: We personally found it challenging because we do so much work around hearing aids with our tinnitus patients we had to sort of re-word everything and personally felt that we weren’t giving the best service that we knew we could offer because we knew the patients would probably benefit from hearing aids in the long term.

Despite a dedicated research training day being provided to all sites, the healthcare professionals indicated that additional research training is needed. Some healthcare professionals found notions of equipoise and randomisation difficult to accommodate within their clinical practice and lacked ‘research confidence’.

Professional 7: It’s hard, it’s very hard and I go to the trial management meetings and we talk about equipoise and we know we are not in equipoise for this because we know that hearing aids work and you have to put on your actors face and say we don’t have the evidence to proves this works we know that in some cases it does work but we don’t have the evidence to prove it, would you be willing to be part of the trial to do that.

Professional 3: I knew very, very little about research and I’d say when as a general clinician we are not that used to doing research so it’s probably a little bit of a daunting task and as well I think it was felt they had a lot of training in the beginning and I could see there was a lot of training going into things and people had done presentations but by the time the study actually got up and running it was quite some time after that.

### Health economics

We report summary statistics self-reported service and device use in Table 4. Further tables summarising unit costings and intensity of service/device use can be found in the Appendix.

**Table 4:** Self-reported healthcare service, device and medication use at 12 weeks

Service	TAU (*n* = 41)				TAU + hearing aid (*n* = 42)			
	**NHS + private**		**Private**		**NHS + private**		**Private**	
	*n*/*N*	%	*n*/*N*	%	*n*/*N*	%	*n*/*N*	%
**Audiologist/hearing therapist**								
Initial assessment	9/28	32·1%	2/9	22·2%	12/32	37·5%	1/9	11·1%
Fitting of hearing aid	9/28	32·1%	1/8	12·5%	14/32	43·8%	1/9	11·1%
Check-up (face to face)	1/28	3·6%	0/1	0·0%	2/32	6·3%	0/2	0·0%
Repair/fixing of hearing aid	2/28	7·1%	0/1	0·0%	3/32	9·4%	0/1	0·0%
ENT doctor	2/27	7·4%	0/2	0·0%	1/31	3·2%	0/1	0·0%
Other	3/3	100%	0/4	0·0%	4/4	100%	1/4	25·00%
**Devices**								
Hearing aid	10/26	38·5%	0/10	0·0%	25/31	80·7%	1/28	3·6%
Sound generator	0/25	0·0%	-	-	0/29	0·0%	-	-
External sound generator	2/26	7·7%	1/2	50·0%	1/31	3·2%	-	-
Mobile phone application	3/27	11·1%	1/3	33·3%	0/31	0·0%	-	-
Other	3/3	100%	0/3	0·0%	1/1	100·0%	1/1	100·0%
**Primary and community**								
General practitioner	3/28	10·7%	1/3	33·3%	3/32	9·4%	0/2	0·0%
Psychologist	0/27	0·0%	-	-	0/31	0·0%	-	-
**Medication**	**NHS**		**Private**		**NHS**		**Private**	
Prescribed	1/28	3·6%	1/28	3·6%	1/31	3·2%	0/32	0·0%

Aggregate service use binary variables (y/n) were amended to 1 or 0 if missing and participants specified individual service contact or no individual service use respectively. If binary variables declared no aggregate service use, individual service use contacts were set to 0 if missing and no other individual service use was observed. “-” corresponds to no observations. It should be emphasised that the observation numbers of private service use are a subset of all providers reported in left-hand columns

The questionnaires were well completed for those which were returned. The TAU group returned 28/41 (68.3%) of the questionnaires, of which 26/28 (93%) provided complete case data on healthcare resource use. A larger number were returned in the TAU plus hearing aids group 33/42 (78.6%), corresponding to 29/33 (88%) complete cases.

The non-complete cases had responded to almost all individual questions, and completion of item level responses were balanced between groups. Participants in both arms reported of receiving an initial assessment and fitting of hearing aids with an audiologist/hearing therapist and to a lesser degree of receipt of hearing aids themselves. Two participants in each of the groups specified receipt of private healthcare services, and one in each group reported receipt of medicines (either NHS or privately prescribed). The resource burden of treating this population largely falls upon the NHS. Costs for received private services and devices were very poorly completed and collection of such items needs refinement going forwards.

Table 5 presents the results of the within-trial economic evaluation. The TAU plus hearing aids group self-reported healthcare costs were significantly higher than the TAU group: a mean difference of £146.75 (£25.70 to £272.07). Quality-adjusted life years (QALYs) derived from the HUI3 reported a broader distribution than the EQ-5D, appearing more sensitive to the tinnitus population within the trial. Mean differences in QALYs at 12 weeks were positively skewed in favour of the TAU plus hearing aids group, yet did not significantly differ from zero across health state utility instruments: EQ-5D 0.002 (95% CI − 0.006 to 0.009), EQ-VAS 0.006 (− 0.006 to 0.016) and HUI3 0.01 (− 0.007 to 0.029).

**Table 5:** Intention-to-treat quality-adjusted life-years and costs at 12 weeks: TAU vs TAU + hearing aid

Variable	TAU (*N* = 41)	TAU + hearing aid (*N* = 42)	Mean difference
**EQ-5D** (*n*)	(27)	(33)	(60)
Unadjusted	0·196 (0·033)	0·178 (0·053)	− 0·017 (− 0·041 to 0·004)
Baseline adjusted	0·185 (0·179 to 0·191)	0·187 (0·181 to 0·192)	0·002 (− 0·006 to 0·009)
Covariate adjusted	0·186 (0·18 to 0·192)	0·186 (0·181 to 0·192)	0·001 (− 0·007 to 0·009)
**EQ-VAS** (*n)*	(27)	(33)	(60)
Unadjusted	0·199 (0·039)	0·186 (0·046)	− 0·013 (− 0·034 to 0·009)
Baseline adjusted	0·189 (0·181 to 0·197)	0·194 (0·187 to 0·202)	0·006 (− 0·006 to 0·016)
Covariate adjusted	0·19 (0·181 to 0·198)	0·194 (0·186 to 0·201)	0·004 (− 0·008 to 0·016)
**HUI3** (*n)*	(26)	(31)	(57)
Unadjusted	0·141 (0·085)	0·132 (0·076)	− 0·009 (− 0·05 to 0·035)
Baseline adjusted	0·131 (0·119 to 0·142)	0·14 (0·13 to 0·151)	0·01 (− 0·007 to 0·029)
Covariate adjusted	0·132 (0·12 to 0·143)	0·14 (0·129 to 0·15)	0·008 (− 0·01 to 0·022)
**NHS Costs** (*n)*	(18)	(20)	(38)
Unadjusted	104·63 (46·09 to 163·17)	251·38 (145·09 to 163·17)	146·75 (25·70 to 272·07)
Multicentre adjusted	108·64 (28·84 to 188·45)	247·77 (155·61 to 339·92)	139·13 (12·58 to 254·85)

Data are mean (SD), or mean difference (95% CI). EQ-5D, EuroQoL-5D. EQ-VAS, EuroQol Visual Analogue Score. HUI3, Health Utilities Index Mark 3. We report univariate distributions as deriving the joint distributions of complete case data presenting differential observational set sizes would inherently exclude a significant number of observations from the larger (QALY) set. Covariate adjustment included continuous variables for baseline utility and age, and binary variables for sex (male as base) and multicentre effects (site one as base—site six was excluded due to collinearity and so included as base). EQ-VAS estimates were divided by one hundred to aid comparison with the other instruments. Estimates of costs considered all reported service use as NHS applicable, as such reporting a lower *n* to avoid double counting the items which we could not guarantee were not private service use

## Discussion

i) Determining feasibility of a multicentre RCT comparing TAU and TAU plus hearing aids

The trial gathered sufficient quantitative and qualitative data upon which the TMG and TSC was able to base a formal feasibility assessment in accordance with the pre-determined feasibility criteria:

1. Over 80% of the recruitment target was achieved.
2. All sites remained open throughout the duration of the trial recruitment phase, with 3/5 sites achieving or surpassing their recruitment targets. The common denominators across these 3 sites were engaged staff and robust research infrastructure.

The overall trial retention rate at 12 weeks was 73%, with return rates ranging from 61 to 100% between centres. The participant interviews highlighted reasons for non-return that can be addressed.

The TFI, identified as relevant to tinnitus and hearing loss by the majority of participants, also showed that it was responsive to change. Sufficient data was also gathered to establish variability and a minimal clinically important difference (MCID) based on the data for this trial, to aid in the sample size calculation for a randomised controlled trial with the TFI overall score as the primary outcome.

The TSC also noted that safety data were successfully collected and that incidences of adverse events are likely to be low in a definitive trial.

3. Both participants and healthcare professionals expressed a conviction in the importance of the trial and acceptability of the trial design. Any areas of the trial design requiring modifications were clearly articulated, and solutions could be implemented to address all highlighted areas.

At the final results reveal meeting, the oversight committees concluded that a definitive RCT would be both feasible and safe, and providing design optimisations based on the present trial findings, delivery of a full RCT is plausible within a 5-year study period.

ii) Optimising the design of a definitive RCT

Recruitment: ‘Non-aidable hearing loss’ was the main reason for ineligibility reported by the centres. We made an attempt during the trial design phase to standardise the definition of ‘aidable hearing loss’; however, clinicians were not able to come to a consensus as they were influenced by their local guidelines and personal beliefs. In order to better understand the prevalence of this reason given on the screening logs, during the course of the trial participating sites were asked to define what constituted the local audiometric criteria for a ‘non-aidable’ hearing loss. There was a marked variability in audiometric criteria for fitting hearing aids between centres, with some fitting much milder hearing losses than others. The mildest criteria that clinicians were using were hearing loss of 15 dB or more at at least one audiometric frequency up to 8 kHz. The most conservative criteria reported by site were average hearing loss of more than 30 dB at at least three mid-frequencies (0.5, 1, 2, 4 kHz). These criteria were reported to be the generic fitting criteria for hearing loss, rather than specific criteria for patients with hearing loss *and* tinnitus. This variability resulted mainly from differences in hearing aid eligibility criteria imposed by different Clinical Commissioning Groups (CCGs). Those sites, where CCGs left the decisions about hearing aids eligibility to clinicians, tended to fit hearing aids for milder hearing losses.

It is possible that clinics who choose to fit milder losses are influenced by findings such as those from the review by Ferguson and colleagues [48] who concluded that hearing aids are effective at improving hearing-specific health-related quality of life, general health-related quality of life and listening ability in people with mild-to-moderate hearing loss. Regardless of the reason for local criteria, our results showed that clinical practice is highly variable for the population of participants with mild hearing loss. This variability of treatment for those with no to mild losses is reflected in the NICE guidelines for tinnitus published in March 2020, which formulated a research question of effectiveness of hearing aids for people with tinnitus and no perceived hearing difficulties [17].

Further definitive trial development work should involve seeking clinical consensus to design the inclusion criteria that combine audiometric criteria and perceived hearing difficulties to address the population with tinnitus where clinical practice is the most variable. Such an approach might also give participating clinicians more confidence to recruit patients to take part in the trial, randomise them, and target the audiology departments that do not normally provide hearing aids for people with such characteristics. This should also address the issue of lack of equipoise amongst clinicians and excluding large numbers of participants from the trial due to ‘non-aidable hearing loss’.

The interviews suggested that successful recruitment of a future trial will also depend on sufficient flexibility within the protocol to allow for local variations in the patient pathway. It is important that patients can be identified and offered the trial, regardless of their referral pathway, and of minimum impact to the scheduling of routine clinical practice appointments.

There was also a marked variability between centres in respect to recruitment rates. Experience of conducting research and/or the support of dedicated research staff (NIHR Clinical Research Network support staff in these instances) were key to on site trial delivery. As many audiologists do not have experience participating in large RCTs, there is a need for detailed and targeted research training for these healthcare professionals to be factored into the trial in terms of both time and cost. Further training will also improve the quality of information provided to participants, helping the aims and processes of the trial to be clear to all who are randomised.

### Retention and outcome collection

For the definitive trial, since it is the intention to include only the TFI and a reduced number of other PROMs, the questionnaire pack will be shorter, reducing participant burden. It will also be offered in an online format in addition to paper, which some participants may find more convenient. Clinicians recruiting into the trial will receive training to help the participants understand the importance of returning the questionnaire packs, irrespective of adherence to treatment allocation or perceived improvement in tinnitus. Further PPI work will be undertaken to determine acceptable ways of explaining the value of these questionnaires to participants, and in which formats and at what times reminders would be most helpful for this population.

There is a suggestion, but no conclusive evidence, to suggest that hearing aid benefit is associated with increased usage. It is however a methodological challenge to quantify what is adequate usage of a hearing aid, as this could vary from patient to patient, dependent on lifestyle factors. In the HUSH feasibility trial, we looked at compliance as hours of participant-reported use compared to hours of clinician-recommended use. The compliance reported here for the intervention group was similar aids similar to recent published data on hearing aid usage that reported that 30% of patients given hearing aids under-use them, and a further 20% do not use them at all [49]. However, for the 39% of participants who reported using the hearing aid less than the audiologist recommended, we cannot be sure if the participant was using the hearing aid sufficiently for their lifestyle needs. It needs to be considered for a future definitive trial, and hearing trials in general, a better way to both define and record intervention compliance in order to better understand the relationship between benefit and usage, and equally to acknowledge that what is sufficient use may vary from person to person. Alternative ways of monitoring compliance with the intervention could be based on actual hearing aid use in terms of hours rather than broad categories. This could be collected as patient-report or alternatively ‘data logging’ could be used to obtain more information regarding duration of daily hearing aids use. Previous studies have shown the latter method might be preferred, as on average users tended to over-report their daily amount of hearing aid use compared to objective measures [50]. However, collecting data logging may require additional face to face visits, which might not always fit within the standard clinical practice. The more widespread use of the remote options for hearing aids fitting and data logging, due to service adjustments required by COVID-19 pandemics, might open the possibility of routine remote data logging collection, which could address the above issue. However, currently not many departments utilise this option.

Participants in each of the groups specified the receipt of hearing services (initial assessment, fitting, and receipt of hearing aids). This may signal heterogeneity in site practice leading to a misunderstanding of patient care pathways and would not represent the true resource burden to the NHS. Similarly, feedback from clinicians indicated that there is considerable variance in the costs of hearing aids between sites which were not captured by our questionnaires. A definitive trial would benefit from a detailed breakdown of both the initial patient pathways and the costs of hearing aids, by device, across sites. Questionnaires should be further refined for the collection of societal costs, such as private services and productivity losses.

iii) Informing the choice of primary outcome for a definitive trial

As the core outcome set for tinnitus is still under development [51], at the outset of this feasibility trial, there was no consensus on which PROM should be used as the primary outcome in a definitive trial.

Eighty-four percent of patients who returned their 12-week questionnaire agreed or strongly agreed that the TFI was a relevant measure for tinnitus and hearing loss, making it the most highly rated of the PROMs administered in terms of relevance. It also showed the highest value for responsiveness.

There is also ample empirical evidence to support both the development and validation of the TFI, giving confidence for its use as a primary outcome in a definitive RCT. The TFI was developed to be (1) discriminative to provide measures of tinnitus distress, (2) evaluative to provide a responsive measure of treatment-related changes and (3) comprehensive to cover multiple domains of tinnitus severity. The TFI has been evaluated for use in clinical practice and research in the UK [52, 53]. This national validation study showed the TFI and its subscales to have excellent reliability to differentiate between individuals perceived tinnitus impact and valid constructs that were measuring different aspects of tinnitus impact. The MCID for improvement in the clinical population was established as 13 points [30], similar to the MCID of 16 points shown in our study.

The primary purpose of fitting hearing aids is to reduce hearing difficulties and improve communication, which was observed as the greater change from baseline for the HHIE for the hearing aid group in the present study. Improvement in hearing and communication might lead to reduction in stress and anxiety that may be associated with hearing difficulties and in consequence may lead to changes in self-reported measures of tinnitus symptom severity [54]. Moreover, intervention adherence might be higher in those participants who perceived benefit for their hearing, potentially resulting in a perceived increased benefit for their tinnitus. However, this was not explored in the current trial. It is therefore important to include a hearing-related PROM in a definitive trial of hearing aids for tinnitus and hearing loss. This will allow us to better understand the interaction between hearing improvement and improvement in tinnitus.

## Conclusions

The HUSH trial was the first trial to assess the feasibility of conducting a multi-centre randomised controlled trial of effectiveness of hearing aids for tinnitus in UK audiology departments. It demonstrated that a definitive RCT is feasible, providing optimisations based on feasibility findings are introduced into the design of future trials. The design of the definitive trial will need to take into consideration current differences between audiology departments in terms of referral and treatment pathways and hearing aid fitting criteria. Including audiometric criteria in trial eligibility will help to target the population of patients with mild hearing loss for whom clinical practice is most variable. This could address the clinical uncertainties around what constitutes an aidable loss and lack of equipoise around who should be included in trial participation. The TFI appears to be the most suitable measure for use as the primary outcome, supported by other secondary outcome measures that this trial showed were important to people with tinnitus. The defining and recording of compliance will also be a methodological challenge for consideration in all future trials of hearing device usage.

Whilst the results of a future definitive trial cannot be guaranteed based on the current feasibility findings, it is nevertheless likely that such a trial will be a needed step forwards towards patient satisfaction and potential savings to the healthcare system. A recent study showed low patient satisfaction with tinnitus management, resulting in a time consuming and costly ‘revolving door’ system, with about 37% of tinnitus referred more than once to secondary care [55]. A definitive trial could address the above issues either by providing evidence facilitating the provision of hearing aids to patients who currently have variable access to that management option, if shown to be effective, or by shifting research avenues and clinical resources to other potentially more effective management options.

Additionally, as there have been relatively few large-scale RCTs engaging UK audiology clinics, there is a gap in research capacity and skills within NHS hearing services. This trial was a step towards supporting audiologists to become confident researchers. Importantly, audiologist and trialist experiences from HUSH are feeding into collective national efforts to develop widespread and accessible training to boost research skills, knowledge and confidence amongst health care professionals conducting trials within NHS hearing services and informing the design of future trials in these contexts and patient populations. It is hoped that this work which the HUSH trial has influenced will continue to prepare the workforce for the successful delivery of future large-scale RCTs within audiology clinics.

## Supplementary Information

**Additional file 1. **CONSORT 2010 checklist of information to include when reporting a pilot or feasibility trial.

**Additional file 2. **Appendix with additional tables.

## Data Availability

The full trial protocol and all study documentation is available on request. The datasets analysed during this trial will be available to researchers upon request from the NCTU (ctu@nottingham.ac.uk), a minimum of 12 months after publication of this paper. Access to the data will be subject to review of a data sharing and use request by a committee including the CI and sponsor and will only be granted upon receipt of a data sharing and use agreement. Any data shared will be pseudonymised which may impact on the reproducibility of published analyses.

## Abbreviations

CRN: Clinical Research Network
EQ-5D-5L: European Quality of Life Index five level version
GP: General practitioner
HADS: Hospital Anxiety and Depression Scale
HHIE: Hearing Handicap Inventory for the Elderly
HRA: Health Research Authority
HRQoL: Health-related quality of life
HUI3: Health Utilities Index Mark 3
MCID: Minimal clinically important difference
MYMOP2: Measure Yourself Medical Outcome Profile
NCTU: Nottingham Clinical Trials Unit
NHS: National Health Service
NICE: National Institute for Health and Care Excellence
NIHR: National Institute for Health Research
NRES: National Research Ethics Service
PROM: Patient reported outcome Measure
QALY: Quality-adjusted life year
RCT: Randomised controlled trial
TFI: Tinnitus Functional Index
TMG: Trial management group
TSC: Trial steering committee
TAU: Treatment as usual
