# Supplementary material for: Effectiveness and cost effectiveness of digital hearing aids in patients with tinnitus and hearing loss: a randomised feasibility trial (THE HUSH Trial)
Source: Pilot Feasibility Stud. 2022 Nov 3;8:235. doi: 10.1186/s40814-022-01188-9 (PMC9630825; doi:10.1186/s40814-022-01188-9)
Supplement: Supplementary file 2 — Additional file 2. Appendix with additional tables. [file 40814_2022_1188_MOESM2_ESM.docx]

**Appendix tables**

**Table A1: Patient reported outcomes summary by timeline by arm**

| **Outcomes** | **At baseline** | | **At 12 weeks** | | **Changes from baseline** | |
| --- | --- | --- | --- | --- | --- | --- |
|  | **TAU**  **(n=41)** | **TAU+Hearing aid (n=42)** | **TAU**  **(n=28)** | **TAU+Hearing aid (n=33)** | **TAU**  **(n=28)** | **TAU+Hearing aid (n=33)** |
| **TFI overall score**  Mean[SD] | 57.1[21.9] | 57[22] | 48.1[26] | 37.8[19.5] | -5.2[18.8] | -20.4[22] |
| **HHIE**  Mean[SD] | 35.3[23.7] | 38.6[24.6] | 30.6[24.1] | 28.1[21.6] | 0.6[13.6] | -8.7[19.8] |
| **HADS overall**  Mean[SD] | 11.1[7.2] | 12.3[7.7] | 10.7[7.5] | 11.4[7.7] | 1.1[6.2] | -0.8[6.5] |
| **HADS – anxiety**  Mean[SD] | 7.1[4.0] | 8.0[4.7] | 6.6[4.3] | 7.5[4.7] | 0.3[3.6] | -0.3[4.3] |
| **HADS – Depression**  Mean[SD] | 4.1[3.9] | 4.3[3.5] | 4.1[4.3] | 3.8[3.7] | 0.8[3.1] | -0.5[2.7] |
| **MYMOP**  Mean[SD] | 3.7[1.4] | 3.6[1.1] | 3.0[1.5] | 2.6[1.3] | -0.4[1.4] | -1[1.3] |

All data are N (%)’s unless specified

**Table A2: Participants’ view of relevance of PROMs at 12 weeks and responsiveness of PROMs**

|  | **All participants (N=61)** | | | | **GRI responsiveness index for change from baseline at 12 weeks** |
| --- | --- | --- | --- | --- | --- |
|  | Relevant to tinnitus and hearing loss | | | |  |
|  | Strongly agree or agree | Neither agree nor disagree | Strongly disagree or disagree | Missing |  |
| TFI | 51(84%) | 3(5%) | 2(3%) | 5(8%) | -0.90 |
| HADS | 22(36%) | 24(39%) | 10(16%) | 5(8%) | 0.34 |
| MYMOP | 40(65%) | 13(21%) | 2(3%) | 6(10%) | -0.67 |
| HHIE | 46(76%) | 7(11%) | 1(2%) | 7(11%) | -0.54 |

All data are N (%)’s unless specified

GRI: Guyatt’s responsiveness index.

| **Table A3. Unit costings of healthcare services and devices.** | |  |
| --- | --- | --- |
| **Service** | **Source** | **Unit cost (£)** |
| **Audiologist/hearing therapist (per contact)** | NHS Reference Costs 2017/18 |  |
| Initial assessment | CA37A | 101·32 |
| Fitting of hearing aid | AS04 | 101·18 |
| Check up (face to face) | AS08 | 53·55 |
| Repair/fixing of hearing aid | AS11 | 35·34 |
| ENT Doctor | 120 (Outpatient) | 106·65 |
| **Devices (per item)** | NHS Reference Costs 2017/18 |  |
| Hearing aid | AS05 / AS06 | 95·17 |
| **Primary and community (per contact)** | Unit Costs of Health and Social Care 2019 |  |
| General practitioner^1^ |  | 33 |
| Psychologist |  | 96 |
| 1. For an average consultation length of 9.22 minutes; excluding qualification and carbon emission costs, and including direct care staff costs. | | |

| **Table A4. Self-reported intensity of service and device use at point of access at twelve weeks.** | | | | | | |  |  |
| --- | --- | --- | --- | --- | --- | --- | --- | --- |
| **Service** | **TAU (n=41)** | | | | **TAU + hearing aid (n=42)** | | | |
|  | **NHS + private** | | **Private** | | **NHS + private** | | **Private** | |
| **Audiologist/hearing therapist** | n/N | Contacts | n/N | Contacts | n/N | Contacts | n/N | Contacts |
| Initial assessment | 5/9 | 1·2 (0·45) | 2/2 | 1·5 (0·71) | 10/12 | 1·5 (0·53) | 1/1 | 3 (-) |
| Fitting of hearing aid | 4/9 | 1·25 (0·5) | 1/1 | 1 (-) | 10/14 | 1·1 (0·32) | 1/1 | 1 (-) |
| Check up (face to face) | 0/1 | - | 0/0 | - | 1/2 | 1 (-) | 0/0 | - |
| Repair/fixing of hearing aid | 1/2 | 1 (-) | 0/0 | - | 3/3 | 1·33 (0·58) | 0/0 | - |
| ENT Doctor | 0/2 | - | 0/0 | - | 0/1 | - | 0/0 | - |
| **Primary and community** |  |  |  |  |  |  |  |  |
| General practitioner | 2/3 | 2 (0) | 1/1 | 3 (-) | 3/3 | 1·67 (0·58) | 0/0 | - |
| Psychologist | 0/0 | - | - | - | 0/0 | - | - | - |
| **Devices** |  | Number |  | Number |  | Number |  | Number |
| Hearing aid | 7/10 | 1·43 (0·54) | 0/0 | - | 17/25 | 1·59 (0·51) | 0/1 | - |
| Sound generator | 0/0 | - | - | - | 0/0 | - | - | - |
| External sound generator | 0/2 | - | 0/1 | - | 0/1 | - | - | - |
| Mobile phone application | 1/3 | 2 (-) | 0/1 | - | 0/0 | - | - | - |
| Data are mean(SD). The number of devices obtained privately were not requested as part of the participant questionnaires. | | | | | | |  |  |

| **Table A5. Self-reported other healthcare service, device, and medication use, intensity, and private device costs at twelve weeks.** | | | | |
| --- | --- | --- | --- | --- |
| **NHS** | **Treatment allocation** | ***n*** | **Contacts** | **Cost (£)** |
| MRI | TAU | 1 | - | - |
| MRI | TAU + hearing aid | 1 | 1 (-) | **-** |
| Mental health advisor | TAU | 1 | - | - |
| Othoptic eyes double vision | TAU | 1 | 1 (-) | - |
| A&E (migraine symptoms) | TAU + hearing aid | 1 | 1 (-) | - |
| Dentist | TAU + hearing aid | 1 | 1 (-) | - |
| Occupational health advisor (at work) | TAU + hearing aid | 1 | 1 (-) | - |
| **Private devices** |  | *n/*N |  |  |
| Hearing aid | - | 1/1 | 2 | 3 |
| External sound generator | - | 1/2 | - | 35 |
| Mobile phone application | - | 0/3 | - | - |
| **Other private devices** |  | *n* | Number |  |
| Sleep app | TAU | 1 | - | - |
| Sleep aid pillow | TAU | 1 | - | - |
| Inside pillow sound bar | TAU | 1 | **-** | - |
| Hearing aid bluetooth connector | TAU + hearing aid | 1 | 1 (-) | 150 |
| **Prescribed medication** |  |  | Doses |  |
| Mild antidepressant | TAU + hearing aid | 1 | - | - |
| Otomize | TAU | 1 | - | - |
| **Private medication** |  | *n*/N |  |  |
| Paracetamol | TAU | 1/1 | 2 | - |
| Data are mean (SD). We transcribed variable names directly from patient reports, paraphrased in the case of inside pillow sound bar. The costs of private healthcare service use were not requested as part of the participant questionnaire. | | | | |
